# Validity of the Adult Eating Behavior Questionnaire and Its Relationship with Parent-Reported Eating Behaviors among Adolescents in Portugal

**DOI:** 10.3390/nu14061301

**Published:** 2022-03-19

**Authors:** Sarah Warkentin, Alexandra Costa, Andreia Oliveira

**Affiliations:** 1 EPIUnit—Instituto de Saúde Pública, Universidade do Porto, 4050-600 Porto, Portugal; alexandra.costa@ispup.up.pt (A.C.); acmatos@med.up.pt (A.O.); 2 Laboratório para a Investigação Integrativa e Translacional em Saúde Populacional (ITR), 4050-600 Porto, Portugal; 3 Department of Public Health and Forensic Sciences and Medical Education, Faculty of Medicine, University of Porto, 4200-450 Porto, Portugal

**Keywords:** appetite, eating, behavior, weight, appetitive traits, adolescents

## Abstract

The Adult Eating Behavior Questionnaire (AEBQ) is a tool developed in the UK, used in the investigation of appetitive traits in adults and adolescents, and later validated in a number of countries. To date, the validity of the AEBQ has not been tested on Portuguese-speaking popula-tions. The aim of this study was to validate the AEBQ in a sample of Portuguese adolescents. Participants were 4483 13-year-olds enrolled in the population-based cohort study Generation XXI. Appetitive traits were self-reported by adolescents through the AEBQ and parents also reported adolescent eating behaviors. Confirmatory and exploratory factor analyses were conducted. Construct validity was tested through correlations between AEBQ subscales and parent-reported eating behaviors, and linear regressions between AEBQ subscales and adolescent body mass index z-scores were performed. Adequate internal consistency and several associations with parent-reported eating behaviors and measured adolescent body mass index z-scores were found. This study supports the validity of a five-factor AEBQ (Food Responsiveness and Enjoyment of Food; Slowness in Eating; Food Fussiness; Emotional Over- and Undereating) to measure appetitive traits among Portuguese adolescents and provides a convenient and easy-to-use tool to be used in large-scale research.

## 1. Introduction

The Behavioral Susceptibility Theory (BST) was formulated trying to explain the gene-environment interplay in the development of excess weight [1]. It proposes that genetics operate through inherited appetitive traits that confer individual differential susceptibility to the food environment. In this sense, individuals with greater obesogenic appetitive traits, such as higher responsiveness towards foods and lower responsiveness for satiety cues, are more likely to overeat in situations when high palatable foods are available [2]. In order to test the BST, a parent-reported questionnaire was developed 20 years ago by Jane Wardle and colleagues, the Children’s Eating Behavior Questionnaire (CEBQ) [3]. The CEBQ measures eight eating behaviors, namely Food Responsiveness, Enjoyment of Food, Emotional Overeating, Desire to Drink, Satiety Responsiveness, Slowness in Eating, Emotional Undereating, and Food Fussiness. It is the most widely used questionnaire to assess relationships between eating behaviors and environmental factors, such as parental-feeding practices [4] and family’s socioeconomic status [5], the influence of genetics [6,7] besides its effects on health outcomes, such as weight gain [1,8] and cardiometabolic risk [9] in children, and has been validated in many countries, including Portugal [10].

When children age, changes may occur in some of these appetitive traits, which may be due to the greater exposure to the (food) environment and the increasing autonomy in eating [11]. In addition, genetic predisposition to certain appetitive traits seems to be better expressed with increasing age and consequently greater maturity, which may explain the variability in adiposity in the population [2,12]. Little is known about the continuity and stability of eating behaviors from childhood to adolescence. Adolescents’ appetitive traits and weight are largely influenced by social and physical contexts, such as their home environment, peers, school and the neighborhood they live in [13,14]. Additionally, disordered eating behaviors have a high prevalence during adolescence, a period of social changes and important life transitions, which may have long-lasting impacts on health. The investigation of appetitive traits in this age-group, aiming to better comprehend its individual, societal and environmental determinants, using large-scale cohorts, is a high research priority.

In 2016 [15], the CEBQ was adapted into a self-reported questionnaire, the Adult Eating Behavior Questionnaire (AEBQ), first among adults in the UK and then, in 2019 [16], in 11–18-years-old UK adolescents. As the CEBQ, this new questionnaire consists of 35 items, which pertain to eight different eating behaviors. The factor structure of the AEBQ applied to UK adolescents was not the same as in the previous study in UK adults. The new Hunger subscale, which measures individual hunger experience, specially developed in the AEBQ validation study among adults, was omitted in the younger age-group, thus improving model fit [16]. The factor structure without the Hunger subscale was confirmed in other validation studies in adolescents in the US [17] and Poland [18], young adults in Australia [19], and adults in Bulgaria [20] and Mexico [21], while others recently confirmed the eight-factor structure of the questionnaire in adults in China [22] and Canada [23]. To date, the validity of the AEBQ has not been tested on Portuguese-speaking populations. The adaptation and development of culturally-appropriate tools is necessary in order to better comprehend the etiology of appetitive traits within each country.

In light of this, the present study aimed to describe the psychometric properties of the AEBQ, including factor structure, internal consistency, and construct validity through associations between the AEBQ subscales, answered by the adolescent themselves, and parent-reported eating behaviors and adolescent measured Body Mass Index z-score (BMIz).

## 2. Materials and Methods

### 2.1. Study Design and Participants

Participants were adolescents enrolled in the population-based cohort study Generation XXI. Recruitment took place between April 2005 and August 2006, in all public maternities of the metropolitan area of Porto (northern Portugal). Demographic and socioeconomic characteristics, obstetric history and previous personal diseases were collected in the maternity units, within 72 h after delivery, through face-to-face interviews performed by trained interviewers. At baseline, data of 8647 children and 8495 mothers were collected, and following evaluations occurred when children were 4 (86% of participation proportion), 7 (80% of participation proportion), 10 (76% of participation proportion) and 13 years of age (55% of participation proportion—lower than expected due to COVID-19 pandemic). The current study included 4483 adolescents with available data on self-reported eating behaviors (i.e., data on AEBQ) at the 13-years old follow-up.

The study was conducted according to the guidelines of the Declaration of Helsinki and was approved by the University of Porto Medical School/S. João Hospital Ethics Committee (27 April 2005), as well as by the Portuguese Data Protection Authority (Protocol code 5833, approved on 30 May 2011). Written informed consent was signed by parents (or legal guardian) and oral consent was obtained from children. 

### 2.2. Measures

#### 2.2.1. Adolescent Self-Reported Eating Behaviors—AEBQ 

Adolescent self-reported eating behaviors were assessed through the AEBQ (Adult Eating Behavior Questionnaire), a self-reported questionnaire that consists of 35 items which are answered through a 5-point Likert scale, ranging from 1—Strongly disagree to 5—Strongly agree. Items pertain to eight subscales, which can be further categorized as food approach and food avoidant behaviors. These two categories of eating behaviors have been previously associated with BMI, with food approach behaviors showing a positive association with BMI and food avoidant behaviors a negative association [15]. The four AEBQ food approach behaviors are Hunger (e.g., I often feel hungry, 5 items), Food Responsiveness (e.g., I am always thinking about food, 4 items), Enjoyment of Food (e.g., I love food, 3 items) and Emotional Overeating (e.g., I eat more when I’m upset, 5 items). The remaining subscales, described as food avoidant behaviors, are Slowness in Eating (e.g., I eat slowly, 4 items), Satiety Responsiveness (e.g., I often get full before my meal is finished, 4 items), Food Fussiness (e.g., I refuse new food at first, 5 items) and Emotional Undereating (e.g., I eat less when I’m annoyed, 5 items). In accordance with the original scale, five of the items were reverse-scored. The AEBQ measured in the original adolescent UK sample showed an adequate internal consistency (Cronbach’s alpha (α) ranging from 0.66 to 0.80) and test-retest reliability (intra-class correlations ranging from 0.78 to 0.92) [16]. 

The transcultural adaptation of the Portuguese version of the questionnaire (AEBQ-Portugal (P)) begun with the translation of the questionnaire into Portuguese by two Pediatric Nutrition researchers fluent in English who worked together by consensus to produce one translated instrument. A back translation was then made by a native English speaker and Public Health researcher which was blinded to the original version of the AEBQ. After the back-translation, the three researchers who were involved in the translation process checked the discrepancies and found a consensus of the best version of the questionnaire.

#### 2.2.2. Parent’s Reported Adolescent Eating Behaviors—CEBQ

Adolescent eating behaviors at the 13-year follow-up were also assessed through a parent-report questionnaire, the Children’s Eating Behavior Questionnaire (CEBQ) [3], which was previously validated at the 7-year follow-up of Generation XXI [10]. This questionnaire showed good internal consistency at 10 years (α coefficients ranging from 0.76 to 0.84) [24]. Parents or main caregivers were asked to respond to the 35-item questionnaire, which pertain to eight subscales, namely Satiety Responsiveness (e.g., My child leaves food on his/her plate at the end of a meal, 5 items), Slowness in Eating (e.g., My child eats slowly, 4 items), Food Fussiness (e.g., My child is difficult to please with meals, 6 items) and Emotional Undereating (e.g., My child eats less when s/he is upset, 4 items), which are generally labelled as food avoidant behaviors, and Food Responsiveness (e.g., If allowed to, my child would eat too much, 5 items), Enjoyment of Food (e.g., My child loves food, 4 items), Desire to Drink (e.g., My child is always asking for a drink, 3 items) and Emotional Overeating (e.g., My child eats more when annoyed, 4 items), which are generally labelled as food approach behaviors. Answers were given using a 5-point Likert scale, ranging from 1—Never to 5—Always. In accordance with the original scale, five of the items were reverse-scored. Due to the low proportion of missing items (<5%), no imputation procedures were performed.

#### 2.2.3. Sociodemographic and Anthropometric Characteristics

Adolescents were weighted in underwear and without shoes, by trained researchers, using a digital scale and the measure was recorded to the nearest 0.1 kg. Height was also measured without shoes, using a fixed stadiometer to the nearest 0.1 cm. Body mass index (BMI) was calculated and sex- and age-specific BMI z-scores (BMIz) were created. Weight status was then defined as ‘underweight’ for z-scores below -2 standard deviations (SD), ‘normal weight’ for z-scores ≥-2SD and ≤1SD, ‘overweight’ for z-scores >1 and ≤2SD and ‘obesity’ for z-scores above 2SD, according to the World Health Organization (WHO) Growth Standards [25].

Sociodemographic characteristics of the mother, such as age, education and household monthly income were obtained through face-to-face interviews conducted by trained researchers at the 13-years follow-up. Mother’s height and weight were measured at the same follow-up (without shoes) and weight status was classified according to WHO cut-offs [26].

### 2.3. Statistical Analyses

In order to test the factor structure of the AEBQ within the Portuguese adolescents, confirmatory factor analyses using structural equation modelling were performed and three previously suggested models [19,21] were tested, as follows: Model 1 included all 35 items and the eight original subscales; Model 2 included all 35 items and seven subscales, with Hunger and Food Responsiveness items loading together into a single subscale and; Model 3 included 30 items and seven subscales, excluding the Hunger subscale (five items). Goodness of fit statistics, including Comparative Fit Index (CFI), Tucker-Lewis Index (TLI), Root Mean Square Error of Approximation (RMSEA), Standardized Root Mean Square Residuals (SRMR), chi-squared statistics (χ2) and degrees of freedom (df) were described for each model. Values ≥0.90 for CFI and TLI, ≤0.06 for RMSEA and ≤0.08 for SRMR were considered as adequate fit [27,28].

Since the above-described models did not achieve an adequate model fit, exploratory factor analyses were conducted. As subscales (or factors) were hypothesized to correlate, oblique rotation (promax) was used. Factor loadings greater than 0.3 in the correlation matrix were considered in order to retain the item in the factor. A scree plot with all 35 items was additionally examined. Internal consistency of items within each identified factor was tested using Cronbach’s α, and values greater than 0.70 were considered acceptable [29]. Mc Donald’s omega h coefficients (ω) were also calculated to eliminate potential errors in the estimation of internal consistency [30]. Factor eigenvalues, percentage of variance explained by each factor, overall Kaiser-Meyer-Olkin (KMO) and Bartlett’s sphericity p-value were also calculated for the proposed AEBQ-P version.

We tested internal consistency of the CEBQ-P in the current population, with the description of Cronbach’s α and Mc Donald’s ω coefficients for each of the eight CEBQ-P subscales. Construct validity [31] was tested through Pearson’s correlations between the identified factors, through correlations between the AEBQ-P subscales and CEBQ-P subscales and through the investigation of relationships between AEBQ-P subscales and adolescent measured BMIz. For all analyses, linear regression coefficients (β) and 95% confidence intervals (95% CI) were computed. Linear regression assumption checks were performed, by testing homoscedasticity and residuals’ symmetric distribution through Q-Q plots and Cook’s distance, and multicollinearity between independent variables was checked using Variance Inflation Factor, and statistical significance was set at 5%. We describe crude regression coefficients between AEBQ subscales and BMIz (not adjusting for sex and age) given the fact that BMIz is already age and sex specific. Statistical analyses were carried out using Stata/SE version 15 (StataCorp. 2017. Stata Statistical Software: Release 15. College Station, TX, USA: StataCorp LLC).

## 3. Results

Participant characteristics are described in Table 1. Adolescents’ median age was 13.4 years, and a third (33.5%) were classified as having overweight or obesity.

Three AEBQ-P factor structures were tested in confirmatory factor analysis and none of the models achieved adequate model fit (Table 2).

Interpretation of the scree plot supported a five-factor solution (i.e., five factors with eigenvalues >1), so an exploratory factor analysis with a five-factor structure was performed and is described in Table 3.

All items from Food Responsiveness and Enjoyment of Food subscales, plus two items of Hunger subscale (#6: I often notice my stomach rumbling and #32: I often feel hungry), loaded into a unique factor, with adequate internal consistency (α = 0.8321 and ω = 0.8360), and 30% of explained variance. All items from Emotional Overeating, Slowness in Eating, Food Fussiness and Emotional Undereating loaded into the corresponding expected factors. Item #31 (I get full up easily) from the original Satiety Responsiveness subscale loaded into the Slowness in Eating subscale in this new factor structure. The three remaining items from Satiety Responsiveness (#11, #23 and #30) showed factor loadings lower than 0.3, so were not included in any factor. Items #9 and #34 from the original Hunger subscale also did not achieve a factor loading ≥0.3, thus were also excluded from this new factor structure. The final factor structure of the AEBQ-P showed an overall high internal consistency (Cronbach’s α and Mc Donald’s ω coefficients ranging from 0.77–0.89) and an overall high sampling adequacy (overall KMO = 0.8788). 

The AEBQ-P food approach behavior Food Responsiveness + Enjoyment of Food showed an expected positive correlation with the remaining food approach behavior subscale Emotional Overeating (r = 0.44, 95%CI 0.42; 0.47) (Table 4). In addition, Emotional Undereating also showed a weak positive correlation with Emotional Overeating (r = 0.30, 95%CI 0.28; 0.33). The remaining subscales showed a weak correlation with each other. Correlations between the proposed AEBQ-P and parent-reported eating behaviors, measured through the CEBQ-P, are also described in Table 4. The CEBQ-P food approach behaviors Enjoyment of Food, Food Responsiveness, Emotional Overeating and Desire to Drink were positively (however weakly) correlated with AEBQ-P Food Responsiveness + Enjoyment of Food and Emotional Overeating and negatively correlated with the AEBQ-P food avoidant behaviors Food Fussiness, Emotional Undereating and Slowness in Eating subscales (however with very weak and weak correlations). A weaker pattern was found between the four CEBQ-P food avoidant subscales and the AEBQ-P food approach behaviors. The most expected pattern was found for CEBQ-P Slowness in Eating, which showed a weak negative correlation with AEBQ-P Food Responsiveness + Enjoyment of Food and showed positive correlations with AEBQ-P Slowness in Eating and Food Fussiness. Finally, parent-reported CEBQ-P eating behaviors (all with adequate internal consistency—Cronbach’s α ≥ 0.75 and Mc Donald’s ω ≥ 0.76) and their equivalent adolescent-reported traits showed moderate correlations (e.g., CEBQ-P Slowness in Eating and AEBQ-P Slowness in Eating: r = 0.59, 95%CI 0.57; 0.61 and CEBQ-P Food Fussiness and AEBQ-P Food Fussiness: r = 0.66, 95%CI 0.64; 0.67).

Lastly, we explored the associations between the AEBQ-P subscales and adolescent measured BMIz (Table 5). Emotional Overeating showed, as expected, a positive association, with adolescent BMIz and the food avoidant subscales Slowness in Eating and Food Fussiness showed a negative association with adolescent BMIz. Contrary to our expectations, the subscale Food Responsiveness + Enjoyment of Food did not reach statistically significant association with adolescent BMIz (*p* = 0.515).

## 4. Discussion

The present study aimed to explore the structure, consistency and validity of the AEBQ in 13-year-old Portuguese adolescents. Our findings support a five-factor structure of the Portuguese version of the questionnaire, with a total of 30 items that measure two food approach and three food avoidant behaviors, as a valid tool to assess eating behaviors among Portuguese adolescents.

The use of cost-effective and convenient tools such as the AEBQ in population-based studies allows researchers to track the development of appetitive traits that could lead to excessive weight gain and to examine the stability of these traits through infancy (by using tools such as the Baby Eating Behavior Questionnaire—BEBQ [32]), childhood (through the CEBQ [3]), adolescence [16] and adulthood [15] (through the AEBQ). Appetitive traits result from a complex combination between genetic predisposition and biological factors, which are shaped by environmental factors such as the family environment, the cultural context, the peers, etc. [33]. Given all these influences, the development of culturally-appropriate tools to measure these traits in each population is warranted.

As described, the exclusion of the Hunger subscale seems to improve the consistency and validity of the AEBQ in samples of adolescents [16,17,18]. In the current analyses, two out of four items of the Hunger subscale were maintained (#6: I often notice my stomach rumbling and #32: I often feel hungry) and loaded into the same factor as the food approach behaviors Food Responsiveness and Enjoyment of Food. As described by Stevenson et al., individuals may perceive and interpret hunger sensations differently (including components such as visceral sensations, liking and wanting states, and cognitions related to food, hunger and eating) [34], thus being difficult to objectively measure this trait. Hunot-Alexander and colleagues also argue that the measured Hunger items reflect an internal state of hunger, driven by episodic signals, rather than a trait, which is related to longer term energy reserves and is described to be more stable [21]. Taken together, the possible exclusion of this factor in future studies may be considered as an appropriate option. In addition to the exclusion of two items from the Hunger subscale, Satiety Responsiveness was also almost entirely excluded from the proposed AEBQ-P (with exception to #31: I get full up easily, which loaded into Slowness in Eating subscale with a factor loading of 0.346). This was an unexpected finding, since the above-mentioned previous AEBQ validation studies in other populations maintained this factor in the questionnaire. The loading of item #31 into the Slowness in Eating subscale is, however, comprehensible due to the existence of moderate correlation between these two food avoidant behaviors [16,18,19,21]. Previous studies in comparable age-groups have shown that the internal consistency of the Satiety Responsiveness subscale fell below the acceptable threshold (i.e., Cronbach’s α < 0.7) [17,18], which was not observed among adults [15,19,22], suggesting that age may play a role in this particular trait. However, further studies are necessary in order to confirm this hypothesis.

The AEBQ-P food approach behaviors were correlated to each other, as expected. Interestingly, we found that both Emotional Over- and Undereating were positively, however weakly, correlated with each other (r(95%CI) 0.30(0.28; 0.33)). It is noteworthy that in the previous validation study of the CEBQ-P within 7-year-olds from the Generation XXI cohort, these two emotional eating traits, derived from a Principal Component Analysis, loaded into the same factor, indicating a high correlation between them [10]. Likewise, a positive moderate correlation was also observed in the AEBQ validation studies in Mexico [21] and Poland [18]. Eating more or less in response to emotional states may have different effects on weight outcomes [3]. However, in a gene-environment interaction study with a large sample of twins, it has been described that children have an underlying tendency to both emotionally under- and overeat, thus these traits seem to share the same aetiology [7], which may also be observed among adolescents. The positive association between Emotional Overeating and BMIz was also found by others in the UK [15], Mexico [21], Bulgaria [20] and Australia [19]. Emotional Undereating, on the other hand, has shown inconsistent associations with weight (with null [19,21,22] and negative [15,20] associations).

In this study, we assessed adolescent eating behaviors through an adolescent-reported questionnaire and a parent-reported questionnaire, which enables the assessment of multiple perspectives of these behaviors, since these may be biased by individual’s attitudes, personality and internal states [35]. All CEBQ-P food approach behaviors, namely Enjoyment of Food, Food Responsiveness, Emotional Overeating, and Desire to Drink, were positively (however weakly) correlated with adolescent-reported eating behaviors (assessed through the AEBQ-P), with greater correlations between the same subscales (e.g., CEBQ-P Emotional Overeating and AEBQ-P Emotional Overeating). In contrast, CEBQ-P food approach behaviors were negatively correlated with AEBQ-P food avoidant behaviors, such as Slowness in Eating and Food Fussiness. Moderate positive correlations between adolescent- and parent-reported eating behaviors were also found for the food avoidant behaviors, such as Slowness in Eating and Food Fussiness and also Emotional Undereating (however with a weaker strength). These results suggest that parents and adolescents have the same perception of these eating behaviors, which could help in the planning of family-based public health interventions. Since the other AEBQ validation studies did not investigate the association between CEBQ subscales and the AEBQ, we are unable to directly compare results. A few studies tested the construct validity using different eating behaviors: External, emotional and restrained eating behaviors, assessed through the Dutch Eating Behavior Questionnaire [16]. Cognitive restraint, uncontrolled and emotional eating, measured through the Three-Factor Eating Questionnaire [22], all showing weak to moderate correlations with the AEBQ subscales.

The non-significant association between the food approach behavior Food Responsiveness + Enjoyment of Food and weight was unexpected; however, this same pattern was also found in the validation study of the AEBQ in Australia [19], China [22], US [17], Bulgaria [20] and Mexico [21]. Although this result is in contradiction to the original AEBQ validation study among UK adults [15], the relation between the AEBQ eating behaviors and weight was not tested in the study with UK adolescents [16], warranting further studies to evaluate this association. The lack of relationship of these two eating behaviors with BMIz is surprising, given the well-known association between these food approach behaviors and weight status in pediatric populations, measured through the parent-reported CEBQ [8]. As described by Hunot-Alexander and colleagues [21], these divergent findings could be a reflection of the self-report nature of the AEBQ versus the parent-reported nature of the CEBQ, as also confirmed by the weak/moderate correlations between the AEBQ and CEBQ subscales. Individuals affected by excess weight might rather not admit their greater responsiveness and enjoyment towards foods, and parents, on the other hand, may feel less concerned in describing their child or adolescent food approach behaviors. In line with this hypothesis, a sensitivity analysis was performed between the parent-reported CEBQ-P Enjoyment of Food and Food Responsiveness subscales and adolescent weight status categories in the current population. Significant associations were found, namely those adolescents with greater scores on CEBQ-P Enjoyment of Food and Food Responsiveness showed odds of 2.47 (IC95% 2.13; 2.87) and 2.12 (IC95% 1.95; 2.29) of being in the overweight/obesity weight category, compared to the underweight/normal weight category, respectively (data not shown).

Food Fussiness showed to be negatively associated with BMIz among Portuguese adolescents, which corroborates with findings in Chinese [22] and Australian young adults [19], but not in Mexican [21] and British adults [15]. As described in a recent review by Wood et al. [36], fussy eating has been inconsistently associated with adiposity in childhood, with several positive, negative and null associations. This trait could indicate a lower intake of main meals and being fussy about eating certain food groups, such as meat, cheese [37], fruit and vegetables [38]. However, individuals may not be selective with regard to ultra-processed foods [39], sweets, sweetened beverages and desserts [40]. This behavior seems to measure a different food avoidant trait and it is important to mention that it is not protective against excessive weight gain in obesogenic food environments [19]. Additionally, the individual perception of appetitive traits and the interpretation of scales may also be influenced by the cultural background of the population. Given these inconsistencies, further studies are necessary aiming to address the relationship between this specific trait and dietary intake in older age-groups, such as adolescents and adults.

This study has some limitations that need to be considered. Since we used data of an ongoing population-based cohort study, neither a pilot study to test the comprehension of the translated AEBQ in a sub-sample of adolescents, nor a test-retest was performed in order to investigate the reliability of the questionnaire. Additionally, the self-reported nature of both the AEBQ and the CEBQ may also have affected results, due to social desirability bias. As stated above, some traits may not be admitted or may be perceived as undesired by adolescents, such as Food Responsiveness, while other behaviors may be perceived as more favorable, such as Slowness in Eating. Finally, other aspects that may have influenced adolescent eating behaviors were not included, such as restrained eating or dieting behaviors.

In addition to being the first study to test the psychometric properties of the AEBQ in a Portuguese speaking country, strengths also include the large sample size and the use of objectively measured weight and height. The use of the widely-used CEBQ to test construct validity is also a strength of this study, allowing the comparison between adolescent and parent-reported eating behaviors.

## 5. Conclusions

In sum, the present study supports the validity of a five-factor AEBQ-P to measure appetitive traits among Portuguese adolescents and provides a convenient and easy-to-use tool to be used among adolescents in large-scale research. The AEBQ-P appetitive traits showed adequate internal consistency and several associations with parent-reported eating behaviors and measured adolescent BMIz. Additional studies, preferably with a prospective design, are necessary in order to investigate the directionality of associations between appetitive traits measured through the AEBQ and weight among adolescents. The testing of associations between AEBQ traits and behavioral measures of appetite, such as eating in absence of hunger and eating rate, is also warranted.

## Figures and Tables

**Table 1:** Mother and adolescent sociodemographic and anthropometric characteristics at 13 years of age follow-up (*n* = 4483).

Mother Characteristics	
*Education (year)—Md (IQR)*	12.0 (7.0)
*Household monthly income (€)—n(%)*	
≤1000	734 (17.1)
1001–2000	2108 (66.2)
>2000	1449 (33.8)
Adolescent Characteristics	
*Age (year)—Md (IQR)*	13.4 (0.18)
*Sex—n(%)*	
Female	2168 (48.4)
Male	2315 (51.6)
*BMIz—M (SD)*	0.45 (1.18)
*Weight status ^a^—n(%)*	
Underweight (<−2SD)	98 (2.2)
Normal weight (≥−2SD and ≤+1SD)	2884 (64.4)
Overweight (>+1 and ≤+2SD)	1071 (23.9)
Obesity (>+2SD)	428 (9.6)

M: Mean, SD: Standard deviations, Md: Median, IQR: Interquartile range, BMI: Body mass index, BMIz: Body mass index z-score. ^a^ Adolescent weight status categories were defined according to the WHO Growth Standards [25].

**Table 2:** Goodness of fit statistics of three models from a confirmatory factor analysis of the AEBQ, among Portuguese adolescents.

Model	Number of Items (Factors)	Tested AEBQ Structure	CFI	TLI	RMSEA (*p*-Value)	SRMR	χ^2^(df)
Model 1	35 (8)	All original AEBQ items and factors	0.782	0.769	0.075 (*p* < 0.001)	0.139	12,099 (560)
Model 2	35 (7)	All original AEBQ items, H and FR loaded into same factor	0.823	0.811	0.073 (*p* < 0.001)	0.129	9147 (434)
Model 3	30 (7)	30 AEBQ items, H subscale (5 items) excluded	0.814	0.802	0.069 (*p* < 0.001)	0.129	10,444 (560)

AEBQ: Adult Eating Behavior Questionnaire, H: Hunger subscale, FR: Food Responsiveness subscale, CFI: Comparative Fit Index, TLI: Tucker Lewis Index, RMSEA: Root Mean Square Error of Approximation, SRMR: Standardized Root Mean Square Residuals, χ2: chi-square statistic, df: degrees of freedom.

**Table 3:** Items, factor loadings and internal consistency of the AEBQ-P among Portuguese adolescents of 13 years old.

AEBQ-P Items	Variance (Proportio*n* of Explained Variance)	Internal reliability Cronbach’s α (Mc Donald’s ω)	Factors Derived from EFA and Respective Factor Loadings				
			Food Responsiveness + Enjoyment of Food	Emotional Overeating	Slowness in Eating	Food Fussiness	Emotional Undereating
17. Given the choice, I would eat most of the time.	4.63 (30%)	0.832 (0.836)	0.683				
22. I am always thinking about food.			0.682				
3. I love eating.			0.632				
4. I look forward to mealtimes.			0.643				
1. I love food.			0.620				
32. I often feel hungry.			0.591				
13. I often feel hungry when I am with someone who is eating.			0.543				
28. I often feel hungry when I am with someone who is eating.			0.530				
33. When I see or smell food that I like, it makes me want to eat.			0.500				
6. I often notice my stomach rumbling.			0.376				
10. I eat more when I’m upset.	4.39 (28%)	0.842 (0.858)		0.837			
8. I eat more when I’m worried.				0.812			
21. I eat more when I’m angry.				0.751			
16. I eat more when I’m anxious.				0.635			
5. I eat more when I’m annoyed.				0.586			
29. I eat slowly.	2.89 (18%)	0.769 (0.786)			0.8426		
25. I am often last at finishing a meal.					0.8122		
14. I often finish my meal (s) quickly.*					0.6976		
26. I eat more and more slowly during the course of a meal.					0.5692		
31. I get full up easily.					0.3455		
12. I enjoy tasting new foods.*	3.25 (21%)	0.846 (0.850)				0.857	
19. I am interested in tasting food I haven’t tasted before.*						0.836	
7. I refuse new foods at first.						0.747	
2. I often decide that I don’t like a food, before tasting it.						0.679	
24. I enjoy a wide variety of foods.*						0.631	
20. I eat less when I’m upset.	4.36 (28%)	0.886 (0.888)					0.894
18. I eat less when I’m angry.							0.853
27. I eat less when I’m annoyed.							0.787
15. I eat less when I’m worried.							0.778
35. I eat less when I’m anxious.							0.774
Excluded items:							
9. If I miss a meal I get irritable.							
34. If my meals are delayed I get light-headed							
11. I often leave food on my plate at the end of the meal.							
23. I get full before my meal is finished.							
30. I cannot eat a meal if I had a snack just before.							
Overall KMO (Bartlett’s *p*-value)	0.8788 (*p* < 0.001)						

* Items with reversed scoring. AEBQ: Adult Eating Behavior Questionnaire, EFA: Exploratory factor analysis, KMO: Kaiser-Meyer-Olkin. Factor loadings < 0.3 are omitted.

**Table 4:** Descriptives and Pearson’s correlations between the five proposed AEBQ-P subscales and the eight parent-reported CEBQ subscales.

AEBQ-P Subscales	M ± SD	Food Responsiveness + Enjoyment of Food	Emotional Overeating	Slowness in Eating	Food Fussiness	Emotional Undereating
		r (95%CI)				
Food Responsiveness + Enjoyment of Food	2.95 ± 0.64	1	0.44 (0.42; 0.47)	−0.11 (−0.14; −0.08)	0.01 (−0.02; 0.04)	0.19 (0.16; 0.22)
Emotional Overeating	2.16 ± 0.80		1	0.04 (0.01; 0.07)	0.07 (0.04; 0.10)	0.30 (0.28; 0.33)
Slowness in Eating	2.62 ± 0.78			1	0.05 (0.02; 0.08)	0.12 (0.09; 0.15)
Food Fussiness	2.77 ± 0.87				1	0.09 (0.06; 0.12)
Emotional Undereating	2.41 ± 0.94					1
CEBQ-P subscales						
Enjoyment of Food (α = 0.84; Ω = 0.84)	3.00 ± 0.44	0.26 (0.23; 0.29)	0.10 (0.07; 0.13)	−0.17 (−0.20; −0.14)	−0.19 (−0.22; −0.16)	−0.01 (−0.04; 0.02)
Food Responsiveness (α = 0.87; Ω = 0.88)	2.20 ± 0.84	0.33 (0.31; 0.36)	0.20 (0.17; 0.23)	−0.19 (−0.22; −0.16)	−0.06 (−0.09; −0.03)	0.04 (0.01; 0.07)
Emotional Overeating (α = 0.84; Ω = 0.86)	2.10 ± 0.74	0.22 (0.19; 0.25)	0.33 (0.30; 0.36)	−0.05 (−0.08; −0.02)	−0.06 (−0.09; −0.03)	0.13 (0.10; 0.16)
Desire to Drink (α = 0.82; Ω = 0.85)	2.04 ± 0.71	0.15 (0.12; 0.18)	0.15 (0.12; 0.18)	−0.05 (−0.08; −0.02)	0.05 (0.02; 0.08)	0.06 (0.03; 0.09)
Slowness in Eating (α = 0.80; Ω = 0.81)	2.43 ± 0.81	−0.10 (−0.13; −0.07)	−0.01 (−0.04; 0.02)	0.59 (0.57; 0.61)	0.04 (0.01; 0.07)	0.02 (−0.01; 0.05)
Satiety Responsiveness (α = 0.75; Ω = 0.76)	2.60 ± 0.48	0.03 (−0.00; 0.06)	0.08 (0.05; 0.11)	0.21 (0.18; 0.23)	0.13 (0.10; 0.16)	0.09 (0.06; 0.12)
Food Fussiness (α = 0.89; Ω = 0.89)	2.93 ± 0.82	−0.03 (−0.06; 0.00)	0.05 (0.02; 0.08)	0.10 (0.07; 0.13)	0.66 (0.64; 0.67)	0.04 (0.01; 0.07)
Emotional Undereating (α = 0.83; Ω = 0.84)	2.30 ± 0.75	0.09 (0.06; 0.12)	0.18 (0.15; 0.21)	0.09 (0.06; 0.12)	−0.00 (−0.03; 0.03)	0.28 (0.25; 0.30)

M: Mean, SD: Standard deviation, r: Pearson’s correlation, CI: Confidence intervals, AEBQ-P: Portuguese version of the Adult Eating Behavior Questionnaire, CEBQ-P: Portuguese version of the Children’s Eating Behavior Questionnaire, α: Cronbach’s alpha; Ω: McDonald’s omega.

**Table 5:** Generalized linear regression analyses between the five proposed AEBQ-P subscales and BMIz among Portuguese adolescents.

AEBQ-P Subscales	BMIz at 13 Years
	β(95%CI)
Food Responsiveness + Enjoyment of Food	−0.02 (−0.07; 0.04)
Emotional Overeating	**0.07 (0.03; 0.12)**
Slowness in Eating	**−0.27 (−0–31; −0.22)**
Food Fussiness	**−0.07 (−0.11; −0.03)**
Emotional Undereating	0.02 (−0.02; 0.06)

AEBQ: Adult Eating Behavior Questionnaire, CI: Confidence intervals. **Bold** represents statistically significant associations (*p* < 0.05).

## Data Availability

The data that support the findings of this study are available on request from the corresponding author, S.W.
